# Room Temperature Operable Autonomously Moving Bio-Microrobot Powered by Insect Dorsal Vessel Tissue

**DOI:** 10.1371/journal.pone.0038274

**Published:** 2012-07-11

**Authors:** Yoshitake Akiyama, Takayuki Hoshino, Kikuo Iwabuchi, Keisuke Morishima

**Affiliations:** 1 Department of Mechanical Engineering, Osaka University, Suita, Osaka, Japan; 2 Department of Applied Molecular Biology and Biochemistry, Tokyo University of Agriculture and Technology, Fuchu, Tokyo, Japan; 3 Department of Bio-Applications and Systems Engineering, Tokyo University of Agriculture and Technology, Nakacho, Koganei, Tokyo, Japan; Argonne National Laboratory, United States of America

## Abstract

Living muscle tissues and cells have been attracting attention as potential actuator candidates. In particular, insect dorsal vessel tissue (DVT) seems to be well suited for a bio-actuator since it is capable of contracting autonomously and the tissue itself and its cells are more environmentally robust under culturing conditions compared with mammalian tissues and cells. Here we demonstrate an autonomously moving polypod microrobot (PMR) powered by DVT excised from an inchworm. We fabricated a prototype of the PMR by assembling a whole DVT onto an inverted two-row micropillar array. The prototype moved autonomously at a velocity of 3.5×10^−2^ µm/s, and the contracting force of the whole DVT was calculated as 20 µN. Based on the results obtained by the prototype, we then designed and fabricated an actual PMR. We were able to increase the velocity significantly for the actual PMR which could move autonomously at a velocity of 3.5 µm/s. These results indicate that insect DVT has sufficient potential as the driving force for a bio-microrobot that can be utilized in microspaces.

## Introduction

Muscle tissue types have developed through the course of evolution, and they are seen today as prototypical soft actuators. Soft actuators are suited to manipulation of fragile micro-objects like living cells in microspaces. Recently, soft actuators have attracted attention from not only roboticists but also scientists and chemists working with soft materials [1]. Unfortunately, there is no conventional soft actuator technology that matches the balanced performance of living muscle tissues [2], [3]. In particular, the energy conversion efficiency of muscle tissues is more than 35% and the life cycle is more than 10^9^. Both of these values are superior to the values that most artificial actuators can attain. Therefore, living muscle tissues and cells have been of interest as novel microactuators. For instance, the muscle tissues and cells are soft and small, they can contract using only the chemical energy in adenosine triphosphate (ATP), and they can regenerate themselves. Several bio-actuators using mammalian heart muscle cells or skeletal muscle cells have already been reported, e.g. pillar bioactuators [4], [5], a micro heart pump [6], [7], a self-assembled microdevice [8], a muscular thin film [9], and various other Microdevices [10]–[18]. However, these devices require precise environmental control to keep the contractile ability of muscle cells. The medium must be replaced every few days and pH and temperature must be kept around 7.4 and 37°C, respectively.

Tissues and cells of insects are generally robust over a much wider range of living conditions as compared to mammals. We previously proposed utilization of insect dorsal vessel tissue (DVT) and cells as an actuator [19] and we demonstrated a micropillar actuator which worked at room temperature for more than 90 days without medium replacement [20]. Surprisingly, the micropillar actuator could work at 5 to 40°C though its contracting velocity and frequency decreased with lowering of temperature and the actuator was irreversibly damaged at 40°C [21]. We also succeeded in controlling the actuation by electrical pulse stimulation [22] and chemical stimulation using insect hormone [23].

In this paper, we demonstrate an autonomously moving polypod microrobot (PMR) driven by the DVT, which will work autonomously at room temperature without any maintenance for a long time. The PMR is able to work autonomously without any external regulation such as electrical stimulation or cardioactive chemical addition because the DVT contracts spontaneously. At first, a prototype of the PMR was fabricated and evaluated. The contractile force of the whole DVT was estimated using deformation of the microstructure of the prototype. Based on the results obtained from the prototype, we fabricated an actual PMR, calculated its velocity, and compared its movement with that of the prototype.

## Results and Discussion

### Assembly of DVT onto the microstructure

**Figure 1:**
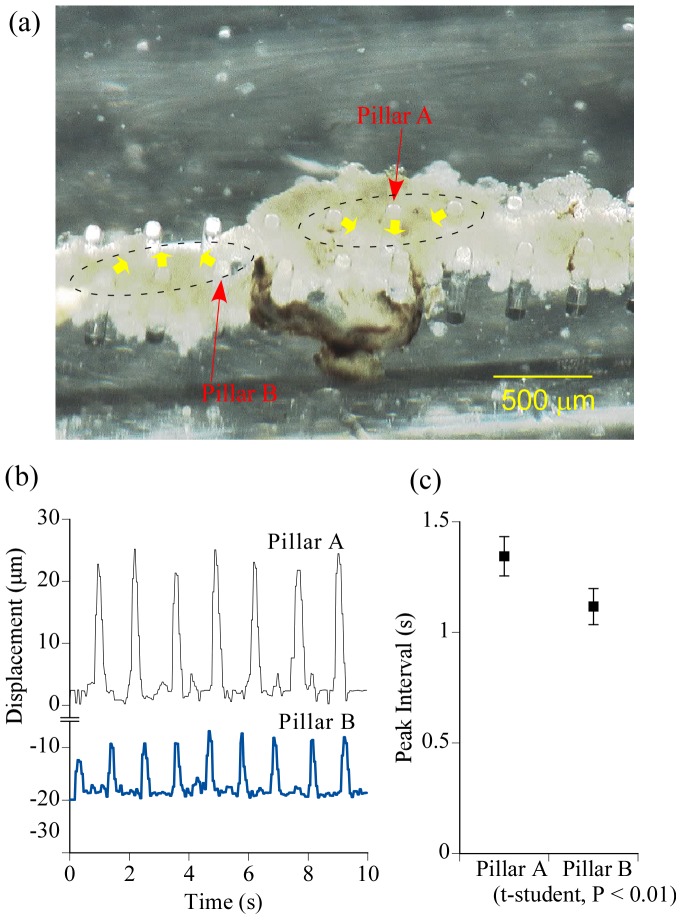
Measurement of displacement of micropillars powered by contraction force of the dorsal vessel tissue. (a) Microscopic image of the prototype PMR. The PMR was not inverted and its base was stuck to the bottom of the petri dish. Micropillars deformed in the direction of the yellow arrows and were divided into two groups (Movie S1 in the SI). The groups are surrounded by the dotted line ellipses. (b) Time courses of deformation of pillars A and B marked in (a). (c) Peak intervals obtained from periods between the contraction peaks marked in (b). There was a significant difference between the peak intervals of pillars A and B.

While the microstructure remained adhered to the bottom of the petri dish, several micropillars of the prototype PMR had been deformed by spontaneous contractions of the DVT for several days after assembling. The prototype used for the experiments had been cultured for 10 or more days because we had previously found that the micropillars were actuated by DVT more vigorously when culturing periods continued for 10 to 50 days rather than immediately after assembling [20]. First, the DVT was just put onto the microstructure. However, the DVT often did not contract in synchronization in spite of forming an organ *in vivo*. An unsynchronized example is shown in Fig. 1 and Movie S1 in the Supporting Information (SI), in which two groups of micropillars were deformed at different frequencies by the same DVT. After several trials, we found it was important that the DVT be kept under some tension. The DVT shrank immediately after excision, and the whole DVT usually contracted in synchronization when the length of the DVT was kept around 10 mm. An average inchworm length was approximately 20 mm. Subsequently, we assembled the DVT onto the microstructure (length, around 10 mm) by tacking two ends of the DVT onto the micropillars under some tension. The mid-region of the DVT which was not stuck to the micropillars contracted freely. The motions consisted not only of shortening along the length of the DVT, but also twisting (Movie S2 in the SI). The latter would seem to be caused by the DVT being assembled under a half or more turn-twisted state. On the other hand, the DVT freely floating in medium was also contracting with twisting. We thought this was because muscle fibers of the DVT aligned helically to the length, not along it.

### Estimation of contractile force and moving velocity of the prototype PMR

**Figure 2:**
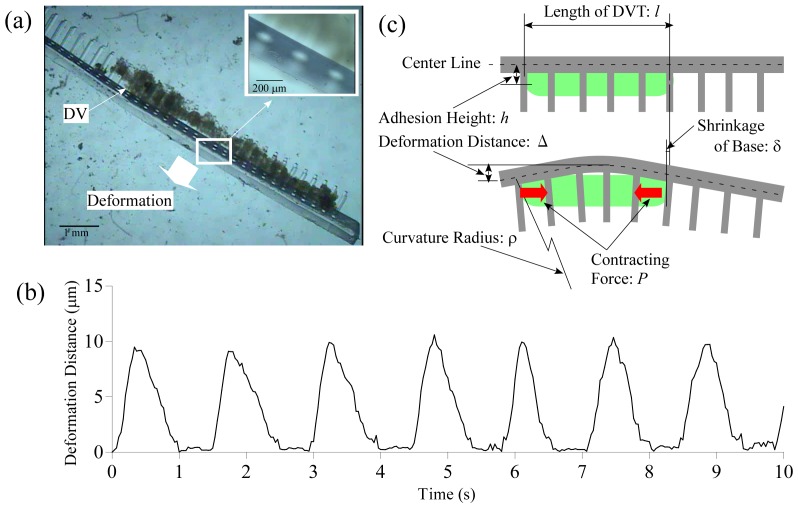
Measurement of deformation distance of the PMR base powered by contraction force of the dorsal vessel tissue. (a) Microscopic images of the prototype from the side. The inset shows an enlarged view of the area surrounded by the white rectangle in the main picture. Movies showing the whole prototype and the inset area, respectively, are available as Movies S3 and S4 in the SI. (b) Time course of deformation distance of the base. The graph was obtained by image analysis processing of Movie S4. (c) The model to estimate the contractile force of the DVT.

The contractile force *P* of the DVT was estimated from the deformation of the base of the prototype. After culturing for 30 days, the prototype was put on its side using tweezers in order to observe the base better. The base was bent by shortening the whole DVT along its length (Fig. 2(a)). The time course of the deformation distance of the base is shown in Fig. 2(b). The base was deformed at 0.78 Hz on average with a standard deviation of 0.19 Hz and the deformation distance was 9.9 µm on average with a standard deviation of 0.50 µm.

The model for the force estimation is shown in Fig. 2(c). Curvature radius *ρ* can be expressed as
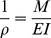
where 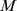 is bending moment 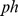 (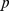: contractile force of the DVT, 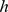: adhesion height from the center line of the base), *E* is Young's modulus for PDMS which is 2.5 MPa [25], and *I* is geometrical moment of inertia 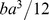 (width of the base 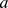, 0.90 mm; height of the base 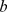, 0.30 mm). By substituting 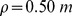 calculated from the length of the DVT (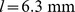) and deformation distance (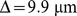), we obtained the contractile force 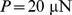 and the shrinkage of the base 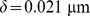. The obtained contractile force was much larger than the force, 4.7 µN, gotten in our previous report [20]. This was because the present value expressed the contractile force of the whole DVT while the value in the previous study expressed the contractile force of a segment of the DVT. The shrinkage of the base was so small that we could not observe it with an optical microscope and the prototype could hardly move. If all the shrinkages were utilized to move, the prototype would move at a velocity of 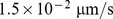.

### Evaluation of prototype PMR

**Figure 3:**
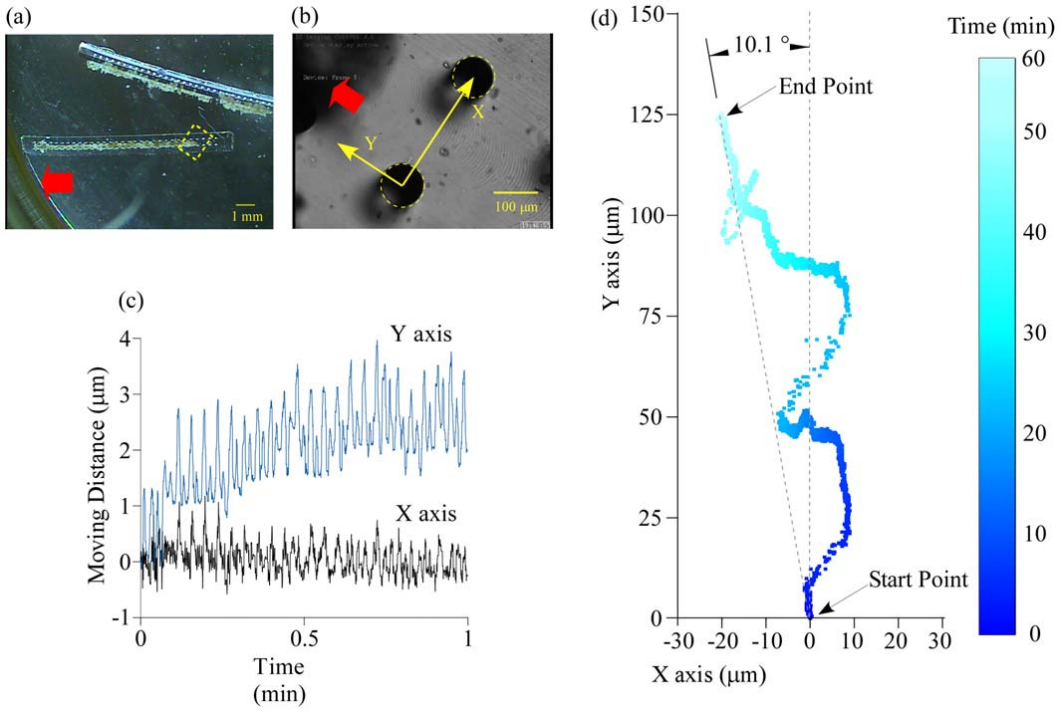
Trajectory measurement of the prototype PMR powered by contraction force of the dorsal vessel tissue. (a) An image of the whole prototype after inversion. (b) Enlarged view of the area surrounded by the dotted rectangle marked in (a). A movie of this area was taken for 60 min (Movie S5 in the SI) and used for image analysis. The moving direction was set as the Y axis and the axis perpendicular to the Y axis was set as the X axis. In (a) and (b), the red arrows show the designed moving direction. (c) Time courses of moving distances along X and Y axes for 1 min. (d) Trajectory of the prototype for 60 min. Time is expressed as gray scale (intensity of blue in the online version). The moving distance from the start point to the end point was 125.5 µm.

The prototype PMR was inverted and observed under zoom microscopy (Figs. 3(a) and (b)). Movie S5 in the SI shows motion in the area of Fig. 3(b) for 60 min with a frame rate 300 times as fast as normal. The prototype moved in the designed direction though the velocity was low. The movements for 1 min along the X and Y axes were analyzed at 30 frames per second (fps) (Fig. 3(c)). The prototype moved along the designed direction (Y axis), namely the moving distance along the perpendicular axis to the Y axis (X axis) was almost zero. The prototype moved not just forward but also swayed back and forth and from left to right; however, overall the prototype moved forward. The swaying oscillations along the X axis and Y axis were approximately 1 µm and 2 µm, respectively. The swaying oscillations along the Y axis were much larger than the shrinkage of the base (0.021 µm) seen in the previous section and there was no mechanism to cause swaying in the direction along the X axis. These results indicated that the prototype was floating and sliding while moving, and not always attached on the bottom of the petri dish.

Next, the movement for 60 min was analyzed at 1 fps and the trajectory is shown in Fig. 3(d). The prototype did not move straightly; it moved forward while shifting from right to left though the overall movement was forward. After 60 min, the prototype had moved 125.5 µm and was on a line inclined 10.1° from the Y axis. The velocity calculated from this result was 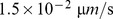, which was 2.3 times as fast as the one estimated from the model of Fig. 2(c). These results suggested that the prototype moved not only by shrinkage of the base but also by sliding while the base deformed and relaxed; that agreed with the presumption in the above paragraph. Though the prototype moved in almost a straight line, there was a small declination between the actual moving direction and the Y axis. The declination could be caused by imbalance of the contractile force of the DVT because the DVT adhered to micropillars which were not precisely on the center line of the base.

### Design and evaluation of the actual PMR

**Figure 4:**
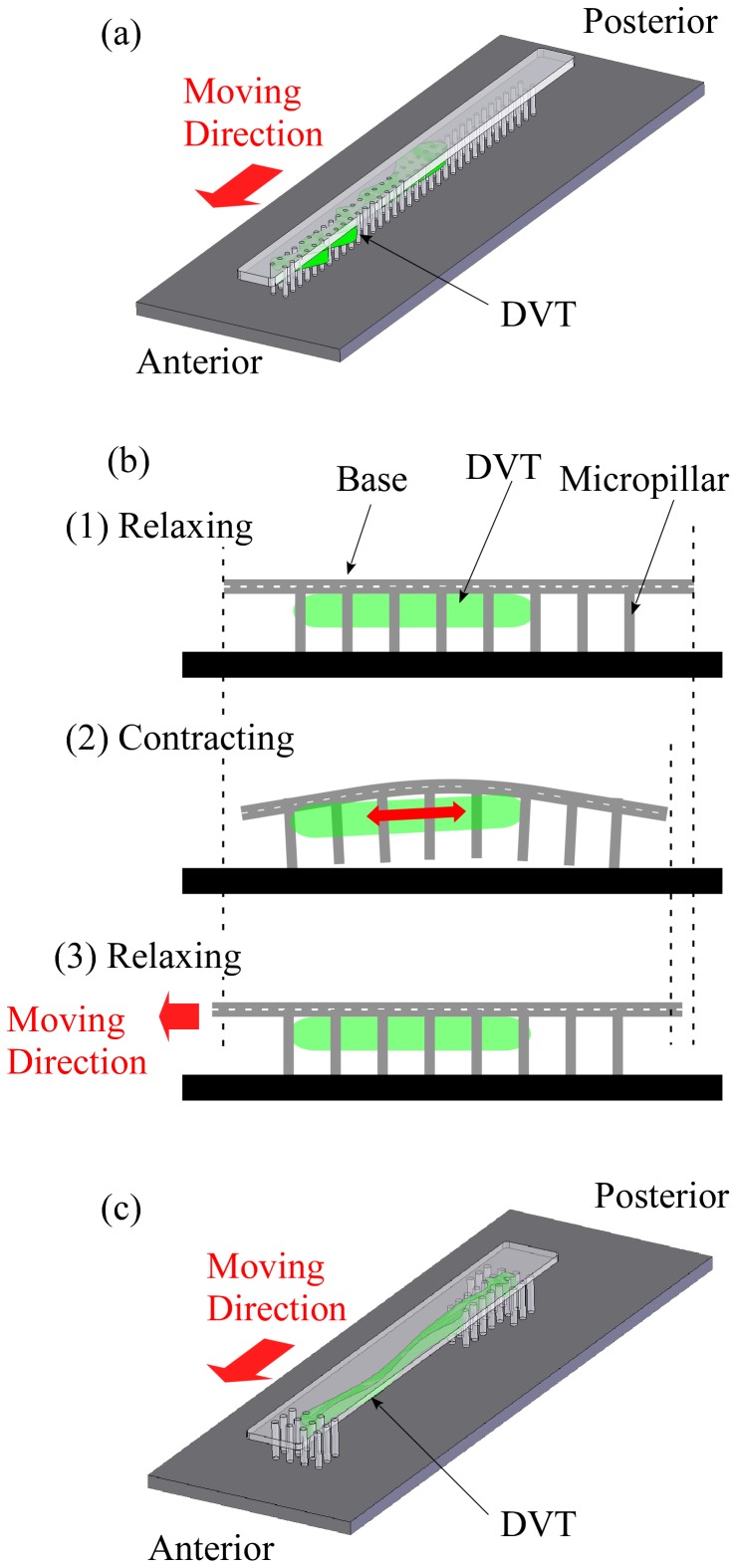
Principle of the prototype Polypod Microrobot (PMR). (a) Schematic illustration of the prototype PMR. (b) Principle of the PMR movement in a lateral view. (c) Schematic illustration of the actual PMR.

**Figure 5:**
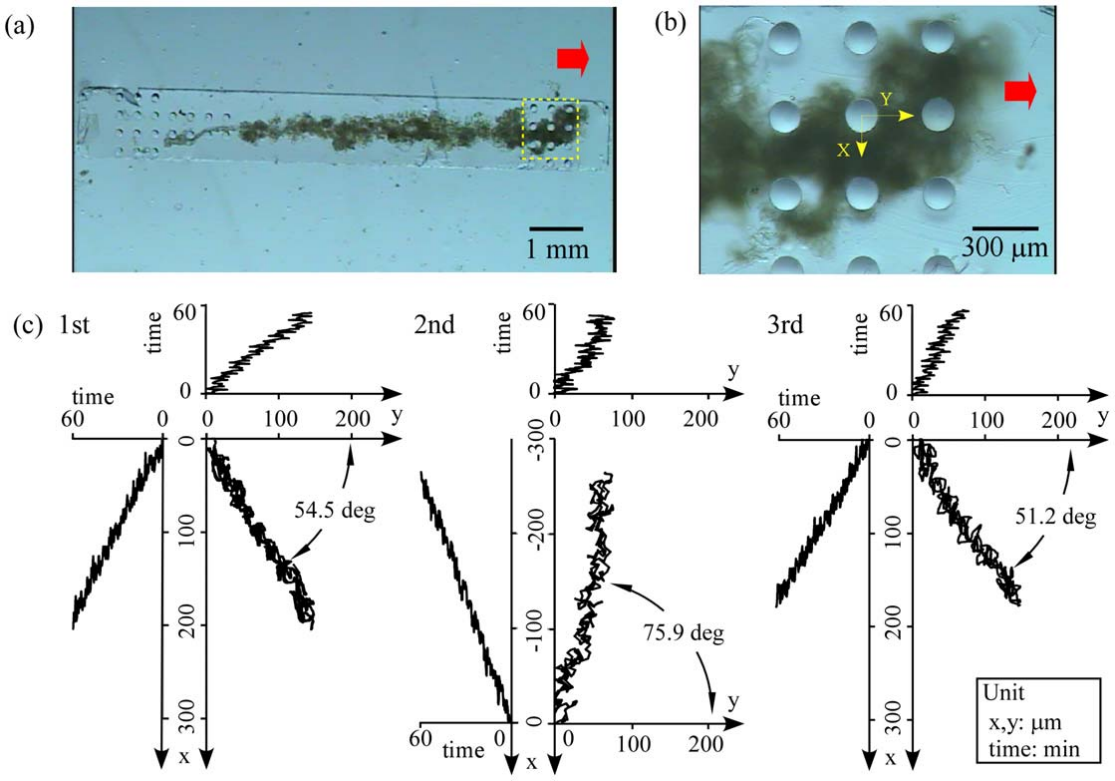
Trajectory measurement of the actual PMR powered by contraction force of the dorsal vessel tissue. (a) An image of the whole actual PMR after inversion. (b) Enlarged view of the area surrounded by the dotted rectangle marked in (a). A movie of this area was taken for 1 min (Movie S6 in the SI) and analyzed with image analysis. The moving direction was set as the Y axis and the axis perpendicular to the Y axis was set as the X axis, the same as for the prototype. In (a) and (b), the red arrows show the designed moving direction. (c) Trajectories and time courses of moving distances along X and Y axes of the prototypes for the 1 min movie period. The moving distances in the first, second, and third experiments were 199, 256, and 175 µm, respectively.

The PMR design was improved (Fig. 4(c)) based on the results obtained by the prototype. To increase the shrinkage of the base, the base thickness was reduced to 0.20 mm, the micropillars except for several around both ends were removed, and the diameter of the remaining micropillars was thickened to 150 µm. The base was widened to 1.35 mm to prevent the actual PRM from toppling over since the prototype PRM often fell over during experiments. Assuming that the other parameters were the same as those of the prototype, we estimated the deformation distance and shrinkage of the base as 20 µm and 0.083 µm, respectively. The shrinkage would increase 4.9-fold, and that would improve the velocity. After 10 days of culturing, the PMR was inverted and observed by zoom microscopy for 1 min (Figs. 5(a) and (b) and Movie S6 in the SI).

In all we fabricated three actual PMRs and evaluated them. Their trajectories and time courses of moving distances along the X and Y axes for the 1 min movie period are shown in Fig. 5(c). We confirmed that the PMRs moved autonomously using spontaneous DVT contractions with the average velocity of 3.5 µm/s (standard deviation of 0.69), which was 100 times faster than the prototype PMR. These results indicate that the DVT will be suitable for wide utilization as a driving force for an autonomously moving microrobot.

Though the velocity was improved much more than had been estimated, the PMRs were on a line that was inclined significantly from the Y axis. The average of the absolute declination angle was 60.4° with a standard deviation of 13.5. Namely, the moving distance along the X axis was larger than that along the Y axis. The PMRs in the first and third experiments yawed to the positive direction of the X axis and the one in the second experiment yawed to the negative direction of the X axis, on the other hand, all the PMRs moved to the positive direction of the Y axis, which is the designed moving direction. The results showed that the imbalance of the contractile force of the DVT could affect the declination direction as the stability of the PMR perpendicular to the moving direction was low due to its symmetrical narrow shape with respect to the moving direction. In addition, since the geometrical moment of inertia was decreased by thinning the base of the microstructure, the declination became much more than that of the prototype.

The shape and size of this DVT-driven microrobot are limited; the size cannot be reduced further as the whole DVT is used as is. Realization of a microrobot with an unconstrained shape needs establishment of a tissue reconstruction technique for the DVT. A dissociation method for DVT using a digestion enzyme has not been established yet and we are working on this problem. Furthermore, toward the goal of fabrication of an insect muscle cell sheet, we have already developed a temperature-responsive culture dish for insect cells by which we decreased the lower critical solution temperature (LCST) from 32 to 15°C [26]. Along with environmental robustness, this has been one of the most important issues in controlling bio-microrobots. Electrical stimulation [16], [17], [22] and chemical stimulation [15], [23] are well-known to regulate muscle contractions. Recently, an optogenetic excitation control technique was reported that expressed a light-activated cation channel channelrhodopsin-2 on mouse cardiomyocytes [27] and on dorsal vessel of *Drosophila melanogaster* [28]. This technique has enabled us to regulate contractions of muscle tissue and cells with high temporal and spatial resolutions. By combining these fabrication and stimulation techniques, a controllable bio-microrobot with the desired size should be created, which would be suited to manipulation of small fragile objects such as biological cells and microfabricated components in enclosed microspaces.

## Materials and Methods

### Designs of prototype and actual PMRs

A prototype of the PMR was fabricated by assembling the DVT onto a microstructure which was an inverted two-row micropillar array consisting of 72 micropillars, each 100 µm in diameter (Fig. 4(a)). The length and width of the base were 12.5 mm and 0.90 mm, respectively. The DVT was located anteriorly to get different anterior and posterior friction forces between the micropillars and the bottom surface. The design principle of movement using anisotropic friction is illustrated in Fig. 4(b). When the DVT shortens its length, the base of the microstructure will arch and shrink almost equally on both sides. On the other hand, when the DVT relaxes and the microstructure returns to the initial shape, the center of gravity of the prototype will be located posteriorly. Therefore, the friction force between the anterior micropillars and the bottom surface will be smaller than the one between the posterior micropillars and the bottom. Then, the anterior micropillars will slip and the posterior micropillars will stick. At this point, the prototype may float slightly since the density of polydimethylsiloxane (PDMS) used to build the microstructure is close to that of the culture medium. The DVT keeps contracting and relaxing autonomously. As a result, the prototype will move to the designed direction at a low velocity. Based on the results obtained from the prototype, we redesigned the PMR to improve the velocity; we refer to this as the actual PMR (Fig. 4(c)).

### Preparation of DVT

DVTs were prepared and cultured as described previously [20]. Briefly, inchworms, Lepidopteran larvae *Ctenoplusia agnata*, were bred and raised at 25°C on an artificial diet. Their DVTs were excised under stereomicroscopy after surface sterilization in 70% ethanol solution [24]. The excised DVTs were kept in TC-100 medium (Sigma-Aldrich, St. Louis, MO) supplemented with 10% fetal bovine serum (FBS) and 1% penicillin-streptomycin solution (Invitrogen Corp., Carlsbad, CA) at 25°C until assembled onto the microstructure.

### Fabrication process of PMRs

The microstructures for the prototype and the actual PMRs were fabricated by molding PDMS (Sylpod184, Dow Corning Toray Co., Ltd, Japan). Poly(tetrafluoroethylene) sheet (PTFE sheet; thickness, 1 mm) was used as substrate for the mold. The substrate was machined by a machining center (ROBODRILL, FANUC, Yamanashi, Japan). The mold was sandwiched between two slide glasses after filling it with PDMS and this was baked at 90°C for 1 h. After baking, the slide glasses were separated and the microstructures were collected while viewing them under a stereomicroscope (SMZ445, Nikon, Tokyo, Japan). A microstructure was put into a 35 mm diameter petri dish, which was treated with oxygen plasma to hydrophilize the microstructure surface using a quick coater apparatus (SC-701, Sanyu Electron, Tokyo, Japan). After hydrophilization, the microstructure surface, not including the micropillars, was adhered to the bottom of the petri dish so it did not float during the following procedures. The microstructure in the petri dish was next coated with Cell-Tak (BD Falcon, Franklin Lakes, NJ) according to the manufacturer's instruction manual. Culture medium (3 mL) was gently poured into the petri dish and the DVT was placed onto the microstructure by using tweezers. The petri dish including the assembled microrobot was cultured at 25°C after sealing with parafilm to prevent the medium from evaporating.

### Image analysis for evaluation of prototype and actual PMRs

Deformation of micropillars and the base of the prototype PMR and moving distances of the prototype and the actual PMRs were obtained by the following experiments based on image analysis. The prototype and the actual PMRs were observed with a zoom microscope (AZ-100, Nikon) equipped with a CCD camera (WAT-221S, Watec, Tsuruoka, Japan), or a digital zoom microscope (KH-7700, Hirox, Tokyo, Japan). The movies were analyzed with analysis software (DippMotion, Ditect, Tokyo, Japan).

## Supporting Information

Movie S1 **Micropillar actuation by DVT before inversion after culturing for 10 days.** This movie was taken through the digital zoom microscope diagonally from the top right.
(MOV)

Movie S2 **Twisting contractions of DVT with both ends fixed to micropillars.**
(MOV)

Movie S3 **Deformation of the base of the prototype PMR at low magnification.** The deformation of the base caused by DVT can be observed.
(MOV)

Movie S4 **Deformation of the base of the prototype PMR at high magnification.** **This** movie was used to measure the deformation distance of the base.
(MOV)

Movie S5 **Microscopic movie of the prototype PMR for 60 min at a play speed 300 times as fast as normal.**
(MOV)

Movie S6 **Microscopic movie of the actual PMR for 1 min at a play speed 6 times as fast as normal.**
(MOV)
